# Characterization of the First Alternavirus Identified in *Fusarium avenaceum*, the Causal Agent of Potato Dry Rot

**DOI:** 10.3390/v15010145

**Published:** 2023-01-02

**Authors:** Xiaofang Zhang, Chunyan Wu, Huihui Hua, Qingnian Cai, Xuehong Wu

**Affiliations:** College of Plant Protection, China Agricultural University, Haidian District, Beijing 100193, China

**Keywords:** *Fusarium avenaceum*, *Fusarium avenaceum* alternavirus 1 (FaAV1), biological characteristics, horizontal transmission, transfection

## Abstract

A novel virus with a double-stranded RNA (dsRNA) genome was isolated from *Fusarium avenaceum* strain GS-WW-224, the causal agent of potato dry rot. The virus has been designated as *Fusarium avenaceum* alternavirus 1 (FaAV1). Its genome consists of two dsRNA segments, 3538 bp (dsRNA1) and 2477 bp (dsRNA2) in length, encoding RNA-dependent RNA polymerase (RdRp) and a hypothetical protein (HP), respectively. The virions of FaAV1 are isometric spherical and approximately 30 nm in diameter. Multiple sequence alignments and phylogenetic analyses based on the amino acid sequences of RdRp and HP indicated that FaAV1 appears to be a new member of the proposed family Alternaviridae. No significant differences in colony morphology and spore production were observed between strains GS-WW-224 and GS-WW-224-VF, the latter strain being one in which FaAV1 was eliminated from strain GS-WW-224. Notably, however, the dry weight of mycelial biomass of GS-WW-224 was higher than that of mycelial biomass of GS-WW-224-VF. The depth and the width of lesions on potato tubers caused by GS-WW-224 were significantly greater, relative to GS-WW-224-VF, suggesting that FaAV1 confers hypervirulence to its host, *F. avenaceum*. Moreover, FaAV1 was successfully transmitted horizontally from GS-WW-224 to ten other species of *Fusarium*, and purified virions of FaAV1 were capable of transfecting wounded hyphae of the ten species of *Fusarium*. This is the first report of an alternavirus infecting *F. avenaceum* and conferring hypervirulence.

## 1. Introduction

Mycoviruses are widely distributed in all major groups of phytopathogenic fungi. Many mycoviruses confer discernable phenotypic changes in their host fungus, including reducing or increasing virulence, referred to as hypovirulence or hypervirulence, respectively [1]. Most mycoviruses are segmented, double-stranded RNA (dsRNA) genomes packed in a non-enveloped, isometric virus-like particle. Mycoviruses with dsRNA genomes belong to the families *Birnaviridae*, *Chrysoviridae*, *Megabirnaviridae*, *Quadriviridae*, *Partitiviridae*, *Picobirnaviridae*, *Reoviridae*, and *Totiviridae*, and the genus *Botybinavirus*; however, some mycoviruses with a dsRNA genome remain unassigned [2,3,4,5,6].

At least forty-seven fully-sequenced mycoviruses have been identified in *Fusarium*, including *Fusarium* incarnatum alternavirus 1 (the proposed family Alternaviridae) [7], *Fusarium* oxysporum ourmia-like virus 1 (*Botourmiaviridae*) [8], *Fusarium* graminearum virus China 9 (*Chrysoviridae*) [9], *Fusarium* graminearum deltaflexivirus 1 (*Deltaflexiviridae*) [10], *Fusarium* poae dsRNA virus 3 (the proposed family Fusagraviridae) [11], *Fusarium* graminearum virus 1 (*Fusariviridae*) [12], *Fusarium* graminearum gemytripvirus 1 (*Genomoviridae*) [13], *Fusarium* pseudograminearum megabirnavirus 1 (*Megabirnaviridae*) [14], *Fusarium* boothii mitovirus 1 (*Mitoviridae*) [15], *Fusarium* equiseti partitivirus 1 (*Partitiviridae*) [16], *Fusarium* redolens polymycovirus 1 (*Polymycoviridae*) [17], *Fusarium* asiaticum victorivirus 1 [18] (*Totiviridae*), and others.

The family Alternaviridae was proposed by Hammond et al. in 2018 [19]. To date, eleven mycoviruses have been designated as alternaviruses in the proposed family Alternaviridae. These include Aspergillus mycovirus 341 (AsV341) [19], *Alternaria alternata* virus 1 (AaV1) [20,21], Aspergillus foetidus virus (AfV) [22], *Fusarium* poae alternavirus 1 (FpAV1) [23], *Fusarium* graminerarum alternairus 1 (FgAV1) [24], Aspergillus heteromorphus alternavirus 1 (AheAV1) [25], *Fusarium* incarnatum alternavirus 1 (FiAV1) [7], *Fusarium* oxysporum alternavirus 1 (FoAV1) [26], *Fusarium* solani alternavirus 1 (FsAV1) [27], Cordyceps chanhua alternavirus 1 (CcAV1) [28], and Stemphylium lycopersici alternavirus 1 (SlAV1) [29].

Potato dry rot caused by *Fusarium* is an economically important and destructive disease of potato tubers that occurs under both field and storage conditions, causing crop losses ranging from 6% to 25% worldwide, while losses up to 60% can occur during long-term storage of potato tubers [30,31]. Many species of *Fusarium* have been demonstrated to cause dry rot of potato tubers [30,32,33], with *F. avenaceum* regarded as one of the top four species of *Fusarium* causing potato dry rot [34,35]. Presently, *Fusarium* coeruleum mitovirus 1 (FcoMV1) and *Fusarium* equiseti mitovirus 1 (FeMV1), isolated from *F. coeruleum* and *F. equiseti* (two species of *Fusarium* that cause potato dry rot), respectively [36,37], are the only mycoviruses that have been reported to be present in species of *Fusarium* causing potato dry rot.

In the present study, a novel mycovirus with a dsRNA genome was isolated from *F. avenaceum* strain GS-WW-224, a strain causing potato dry rot, and tentatively designated as *Fusarium avenaceum* alternavirus 1 (FaAV1) in the proposed family Alternaviridae. This is the first alternavirus identified in *F. avenaceum*. The effect of FaAV1 on the phenotype of the host fungus *F. avenaceum* was characterized. Horizontal transmission of FaAV1 from *F. avenaceum* to ten other species of *Fusarium* (including *F. acuminatum*, *F. brachygibbosum*, *F. equiseti*, *F. flocciferum*, *F. graminearum*, *F. oxysporum*, *F. proliferatum*, *F. redolens*, *F. sambucinum*, and *F. solani*) was also evaluated. Moreover, the ability of purified virions of FaAV1 to transfect the hyphae of these ten other species of *Fusarium* mentioned above was studied.

## 2. Materials and Methods

### 2.1. Fungal Strains

Eleven strains representing eleven species of *Fusarium* (Table 1), including *F. avenaceum* strain GS-WW-224 carrying FaAV1, were used in this study. All of these strains were isolated from potato tubers with symptoms of dry rot that were collected from potato production regions in China. The strains were identified based on morphological characteristics, as well as by sequence analysis of the translation elongation factor 1α (TEF-1α) gene and the internal transcribed spacer region of ribosomal DNA (rDNA-ITS) using previously described methods [32,33]. Except for *F. avenaceum* strain GS-WW-224 infected by FaAV1, the ten other species of *Fusarium* were demonstrated to be mycovirus FaAV1-free based on reverse transcription-polymerase chain reaction (RT-PCR) detection of FaAV1 using the FaAV1-specific primers (dsRNA2-gap-1F and dsRNA2-gap-1R, which are listed in Supplementary Table S1). Additionally, a FaAV1-free strain of *F. avenaceum* derived from strain GS-WW-224 was also used in the study. The FaAV1-free strain of *F. avenaceum*, designated as GS-WW-224-VF, was obtained by eliminating the mycovirus FaAV1 from strain GS-WW-224 using the hyphal tipping technique [38,39]. All twelve of the indicated *Fusarium* strains were cultured on potato dextrose agar (PDA) plates in the dark at 25 °C for seven days prior to their use.

### 2.2. Extraction and Purification of dsRNA

*Fusarium avenaceum* strain GS-WW-224 was cultured on PDA plates on top of cellophane film membranes (PDA-CF) in the dark at 25 °C for seven days, and then used to extract dsRNA. Briefly, approximately 1.0 g fresh weight of fungal mycelia was frozen in liquid nitrogen and ground to a fine powder for dsRNA extraction using the CF11 cellulose (Sigma-Aldrich, China) chromatography method as previously described [40]. The extracted dsRNA was then treated with DNase I (TaKaRa, Dalian, China) and S1 nuclease (TaKaRa, Dalian, China) to remove genomic DNA and single-stranded RNA (ssRNA), respectively. The quality of the obtained dsRNA was assessed by electrophoresis on a 1% (*w*/*v*) agarose gel containing Tris-acetate-EDTA (TAE) and ethidium bromide (EB), and then purified using a gel extraction kit (Aidlab Biotechnologies, Beijing, China) according to the manufacturer’s instructions. The purified dsRNA was stored at −80 °C until further use.

### 2.3. Synthesis and Molecular Cloning of Complementary DNA (cDNA)

The synthesis and molecular cloning of cDNA was performed as described previously [41]. Briefly, purified dsRNA was coupled to a tagged random primer, RACE3RT (5′-CGATCGATCATGATGCAATGCNNNNNN-3′), for synthesizing the first strand of cDNA using moloney murine leukemia virus (M-MLV) reverse-transcriptase (TaKaRa, Dalian, China) to perform the reverse transcription (RT). Then, the required random cDNA products were used as templates, which were amplified by polymerase chain reaction (PCR) using a specific primer, RACE3 (5′-CGATCGATCATGATGCAATGC-3′). The resulting PCR products (more than 800 bp in size) were ligated into the pTOPO-TA vector (Aidlab Biotechnologies, Beijing, China), which was then transformed into *Escherichia coli* Top10 cells (Aidlab Biotechnologies, Beijing, China). All positive clones were sequenced and the obtained sequences were used to design mycovirus-specific primers (Supplementary Table S1). These primers were used to fill in the sequence gaps between clones of cDNA by reverse transcription-polymerase chain reaction (RT-PCR). RNA-ligase-mediated rapid amplification of cDNA ends (RLM-RACE) polymerase chain reaction (PCR) amplification method with an RNA adapter, PC3-T7 Loop adapter (5′-p-GGATCCCGGGAATTCGGTAATACGACTCACTATATTTTTATAGTGAGTCGTATTA-OH-3′) and T4 RNA ligase (TaKaRa, Dalian, China) were used [31,32] to clone the 5′- and 3′-terminal sequences of the genome. Then, a PC2 primer (5′-CCGAATTCCCGGGATCC-3′) and sequence-specific primers (Supplementary Table S1, which were designed based on the sequences of proximal regions) were used for the amplification of terminal sequences. Sequencing was performed by Beijing Tianyihuiyuan Biotechnology Co., Ltd. (Beijing, China). The genome sequence was determined using the sequences of at least three independent overlapping clones, and the whole genome sequence was assembled using DNAMAN 7.0 (Lynnon Biosoft, Montreal, Canada).

### 2.4. Sequence Analysis and Phylogenetic Tree Construction

CLUSTAL_X was used to conduct multiple alignments [42]. Putative open reading frames (ORFs) in the dsRNAs were identified using the ORF Finder program (https://www.ncbi.nlm.nih.gov/orffinder/, accessed on 13 December 2022). Phylogenetic trees based on the deduced amino acid (aa) sequences of RNA-dependent RNA polymerase (RdRp) and hypothetical protein (HP) were constructed using the maximum-likelihood (ML) method in MEGA version 6.0 software with a bootstrap value of 1000 [43]. The reference sequences of viruses used to construct the phylogenetic trees were obtained from the National Center for Biotechnology Information (NCBI) (http://www.ncbi.nlm.nih.gov/genomes, accessed on 13 December 2022).

### 2.5. Extraction, Observation and Confirmation of Virions

Extraction and purification of virions of FaAV1 obtained from *F. avenaceum* strain GS-WW-224 were performed as previously described [44,45]. Briefly, *Fusarium avenaceum* strain GS-WW-224 was cultured on PDA plates on top of cellophane film membranes (PDA-CF) in the dark at 25 °C for seven days. The resulting cultures were used to harvest mycelia. Approximately 15.0 g of mycelia were ground to a fine powder in liquid nitrogen and homogenized in 50 mL of pre-cooled 0.1 mol/L sodium phosphate buffer saline (PBS, pH 7.0) at 13,000 rpm for 10 min. The resulting mycelial suspension was then subjected to centrifugation at 10,000× *g* for 20 min. The obtained supernatant was centrifuged again at 20,000× *g* for 2 h at 4 °C, after which the precipitate was suspended in 4 mL 0.1 mol/L PBS. The resulting suspension was then gently loaded onto a sucrose density gradient (10–40%). The suspension was ultra-centrifuged at 105,000× *g* for 2 h, and layered onto 20% sucrose. The pellets were resuspended in 1 mL of 0.05 mol/L PBS (pH 7.0) and purified by equilibrium centrifugation at 100,000× *g* for 3 h at 4 °C. The obtained virions were suspended in 100 µL 0.05 mol/L PBS (pH 7.2), stained with 2% uranyl acetate, and observed under a transmission electron microscope (TEM) (JEM-1230, JEOL, Tokyo, Japan).

To confirm that FaAV1 was responsible for the formation of the virions extracted from *F. avenaceum* strain GS-WW-224 using RT-PCR detection of dsRNA in virions of FaAV1, total RNA was extracted from the virions of FaAV1 using TRIzol Reagent (Invitrogen, CA, USA) according to the manufacturer’s instructions and reverse amplified to synthesize first-strand of cDNA using the methods described in Section 2.3. Then, PCR amplification was performed using FaAV1-specific primers (dsRNA2-gap-1F and dsRNA2-gap-1R, listed in Supplementary Table S1). The resulting PCR product was identified following electrophoresis in a 1.0% (*w*/*v*) agarose gel.

### 2.6. Effect of FaAV1 on the Phenotype of F. avenaceum

Strains GS-WW-224 and GS-WW-224-VF cultured on PDA plates for ten days at 25 °C in the dark were used to observe general colony morphology. Mycelial plugs (5 mm in diameter) were cut from colony margins of seven-day-old cultures of strains GS-WW-224 or GS-WW-224-VF grown on PDA plates at 25 °C in the dark and placed in the center of plates (90 mm in diameter) containing 20 mL PDA. Colony diameter and spore concentration of these two strains were determined after culturing on the PDA plates for seven days at 25 °C in the dark, according to the methods described in a previous report [46].

Mycelial plugs (5 mm in diameter) from seven-day-old cultures of strains GS-WW-224 or GS-WW-224-VF grown on PDA plates at 25 °C in the dark were used to obtain the dry weight of mycelial biomass. The mycelial plugs were placed in a 250 mL conical flask (5 mycelial plugs per flask) containing 100 mL potato dextrose broth (PDB). The fungal hyphae were collected from the flask after culturing for seven days at 25 °C, placed on four layers of cheesecloth, and then washed with sterile distilled water three times to remove any residual medium. The harvested hyphae were placed at 55 °C for 24 h which allowed them to obtain a constant weight, after which the dry weight of mycelial biomass was measured. The experiment comprised three biological replicates, and the experiment was repeated twice. A paired t-test was performed to determine significant statistical differences (* *p* < 0.05; ** *p* < 0.01; *** *p* < 0.001; **** *p* < 0.0001) between strains using Graphpad Prism version 8.0.

### 2.7. Pathogenicity Test

The pathogenicity of strains GS-WW-224 and GS-WW-224-VF was determined as previously described [47] with minor modifications by inoculating healthy potato tubers (cv. Favorita) that were uniform in size (25–30 g per tuber) with spore suspensions adjusted to the concentration at 10^6^ spores/mL. Briefly, potato tubers were first disinfected by immersion in 0.5% sodium hypochlorite solution (NaClO) for 5 min, followed by three washes with sterile distilled water, and then air dried. Potato tubers were wounded (4 mm in width and 10 mm in depth) using a sterile cork borer and then spore suspensions were pipetted into the wounds (10 μL per potato tuber). An equivalent amount of sterile distilled water was added into wounded potato tubers, which served as a control. The treated potato tubers were placed in a sealed plastic box and incubated in a growth chamber at 25 °C and 90% relative humidity in the dark for seven days. The width and depth of lesions on each of the treated tubers were measured after seven days of incubation. The assay utilized three biological replicates, with ten potato tubers per replicate, and the test was repeated twice. A paired t-test was performed to determine significant statistical differences (* *p* < 0.05; ** *p* < 0.01; *** *p* < 0.001; **** *p* < 0.0001) between strains using Graphpad Prism version 8.0.

### 2.8. Horizontal Transmission of FaAV1 and Transfection with Purified Virions of FaAV1

A hygromycin B phosphotransferase gene conferring hygromycin resistance was transformed into strains of ten other species of *Fusarium* (Table 1) using previously described methods [48]. The transformed strains of the ten other species of *Fusarium* carrying the hygromycin B phosphotransferase gene were then used in the horizontal transmission assay of FaAV1 and the transfection test with purified virions of FaAV1.

Using previously described methods [8,49,50], mycelial agar plugs (5 mm in diameter) were obtained from seven-day-old cultures (grown in the dark at 25 °C) of strain GS-WW-224 (donor strain) and each of the ten strains (recipient strains) of the ten other species of *Fusarium* carrying a hygromycin B phosphotransferase gene were grown in the dark at 25 °C and then used in the paired-cultures. A plug of the donor strain and a plug of each of the recipient strains were cultured in close proximity (approximately 40 mm) to each other on the same PDA plate (not amended with 50 µg/mL of hygromycin) for five days at 25 °C in the dark. Each pairing was replicated across three plates. Mycelial blocks from the fungal-paired PDA plates were then transferred to new PDA plates amended with 50 µg/mL of hygromycin and cultured for seven days at 25 °C in the dark. Mycelial transfers that grew on PDA plates containing hygromycin were then transferred to new PDA plates containing hygromycin to purify the derivative strains. Colonies of the derivative strains were subcultured twice on PDA plates containing hygromycin and then used to extract dsRNA using the methods described in Section 2.2. Then, the extracted dsRNA was used to synthesize the first strand of cDNA to perform the reverse transcription (RT) using the methods described in Section 2.3. Gel electrophoretic profiles of dsRNA and RT-PCR detection of dsRNA of FaAV1 using FaAV1-specific primers (dsRNA2-gap-1F and dsRNA2-gap-1R, listed in Supplementary Table S1) were conducted on the derivative strains to determine if FaAV1 had been successfully transmitted from *F. avenaceum* strain GS-WW-224 to the strains of the ten other species of *Fusarium* carrying the hygromycin B phosphotransferase gene.

Purified virions of FaAV1 were filtered through Ultrafree-MC sterile centrifugal filter units (Millipore, Tokyo, Japan) [51] and used in a viral infection assay. Briefly, 10 µL of virion filtrate was placed on the newly-grown hyphae of three-day-old mycelia of strains of the ten other species of *Fusarium* carrying a hygromycin B phosphotransferase gene that were cultured on PDA plates at 25 °C in the dark. Mycelia of the ten other species of *Fusarium* were then gently touched with an inoculation needle to create wounds and facilitate the transfection of virions. The cultures were then incubated for another three days at 25 °C in the dark to obtain newly-generated mycelia. Three mycelia plugs (referred to as regenerants) were randomly selected and grown on top of cellophane film membranes in fresh PDA plates for seven days at 25 °C in the dark. The resulting cultures were used to extract dsRNA of FaAV1 using the methods described in Section 2.2, which was used to synthesize the first strand of cDNA to perform the reverse transcription (RT) using the methods described in Section 2.3. Gel electrophoretic profiles of dsRNA and RT-PCR detection of dsRNA of FaAV1 using FaAV1-specific primers (dsRNA2-gap-1F and dsRNA2-gap-1R, listed in Supplementary Table S1) in the regenerants were conducted to determine if virions of FaAV1 could transfect the hyphae of the ten other species of *Fusarium* carrying a hygromycin B phosphotransferase gene. The colonies of regenerants confirmed to be carrying FaAV1 were subcultured three times to determine the stability of FaAV1 in each subculture.

## 3. Results

### 3.1. Complete Sequence and Phylogenetic Analysis of FaAV1

**Figure 1:**
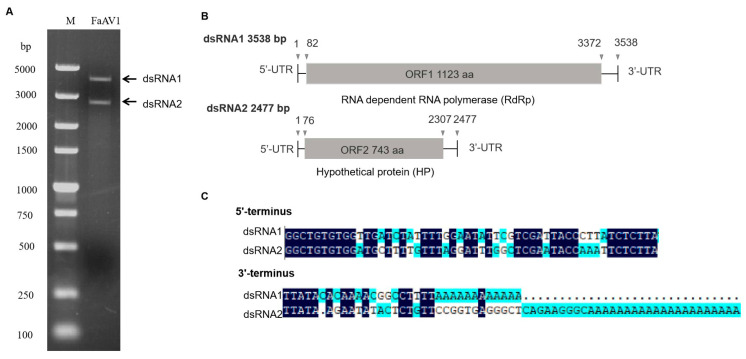
Double-stranded RNAs (dsRNAs) extracted from *Fusarium avenaceum* strain GS-WW-224. (**A**) 1.0% (*w*/*v*) agarose gel electrophoretic profiles of dsRNA extracted from *F. avenaceum* strain GS-WW-224, which was treated with DNase I and S1 nuclease. Lane M: DNA molecular marker DL 5000. (**B**) Schematic representation of the complete genome of *Fusarium avenaceum* alternavirus 1 (FaAV1), with open reading frames (ORFs) and coding regions indicated by gray boxes and triangles, respectively. (**C**) Sequences of the 3’- and 5’- untranslated regions (UTRs) of FaAV1 with identical nucleotides indicated in dark blue.

**Figure 2:**
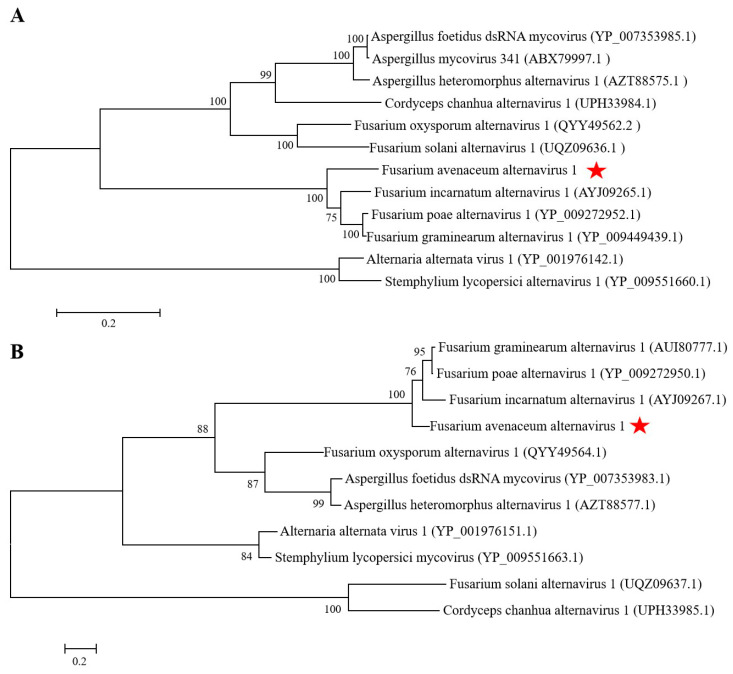
Phylogenetic trees constructed based on the amino acid (aa) sequences of RNA-dependent RNA polymerase (RdRp) and hypothetical protein (HP) of *Fusarium avenaceum* alternavirus 1 (FaAV1) and related viruses in the proposed family Alternaviridae using the maximum-likelihood (ML) method with 1000 bootstrap replicates. (**A**) Phylogenetic tree constructed based on the aa sequences of RdRp. Bar scale represents a genetic distance of 0.2 aa substitutions per site. Bootstrap values greater than 50% are shown above the branches. Red star indicates the position of FaAV1. (**B**) Phylogenetic tree constructed based on the aa sequences of HP. Bar scale represents a genetic distance of 0.2 aa substitutions per site. Bootstrap values greater than 50% are shown above the branches. Red star indicates the position of FaAV1.

The dsRNA extracted from *F. avenaceum* strain GS-WW-224 was electrophoresed in a 1.0% (*w*/*v*) agarose gel and two bands were observed (Figure 1A). The complete nucleotide sequences of the two dsRNAs were determined to be 3538 bp (dsRNA1) with a G + C content of 50.1% and 2477 bp (dsRNA2) with a G+C content of 53.5%, respectively. The genome sequences were deposited in GenBank under the accession numbers OK556479 for dsRNA1 and OK556480 for dsRNA2. The ORF1 in dsRNA1 was found to encode a putative RNA-dependent RNA polymerase (RdRp) of 1123 aa (amino acid) residues, which was 84.42% identical to its counterpart in *Fusarium* poae alternavirus 1 (FpAV1) (Figure 1B and Table 2). The ORF2 in dsRNA2 was found to encode a 743-aa polypeptide with 80.89% identity to a hypothetical protein (HP) encoded by ORF2 in *Fusarium* graminearum alternavirus 1 (FgAV1) (Figure 1B and Table 2). The 5ʹ- and 3ʹ-untranslated regions (UTRs) of the two dsRNAs (dsRNA1 and dsRNA2) were highly conserved and poly-adenylated (Figure 1C). Phylogenetic trees based on the aa sequences of RdRp of FaAV1 and eleven other dsRNA viruses (Figure 2A) and the aa sequences of HP of FaAV1 and ten other dsRNA viruses (Figure 2B) indicated that FaAV1 clustered closely with three alternaviruses, namely *Fusarium* graminearum alternavirus (FgAV1), *Fusarium* incarnatum alternavirus (FiAV1), and *Fusarium* poae alternavirus 1 (FpAV1), all of which contain three dsRNA segments [7,23,24]. Collectively, these results suggest that FaAV1 is a novel member of the proposed family Alternaviridae.

### 3.2. Observation of Virions and Confirmation of FaAV1 Assembling Virions

**Figure 3:**
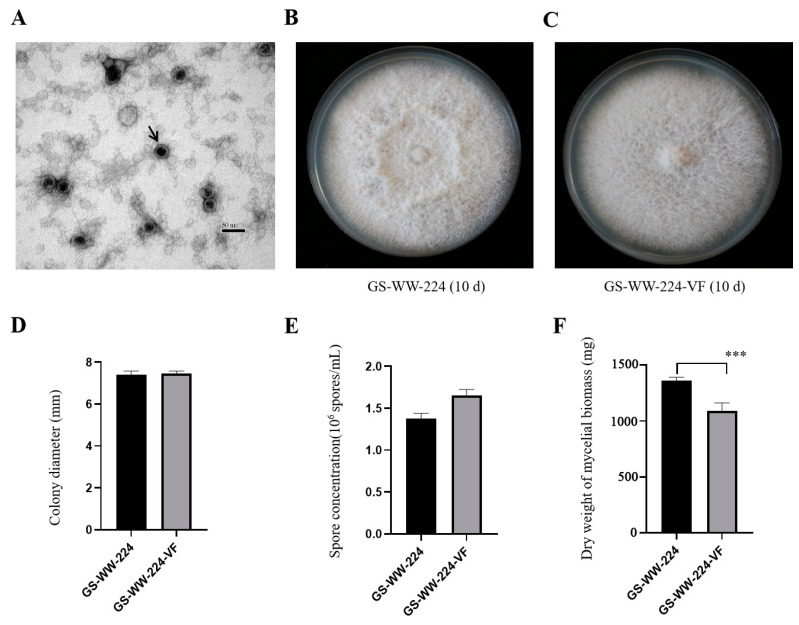
Virions of *Fusarium avenaceum* alternavirus 1 (FaAV1) and colony morphology, colony diameter, spore concentration, and dry weight of mycelial biomass of *Fusarium avenaceum* strains GS-WW-224 and GS-WW-224-VF. (**A**) Transmission electron microscope (TEM) image of virions of FaAV1 indicated by a black arrow. Scale bar = 50 nm. (**B**) Colony morphology of strain GS-WW-224 cultured on potato dextrose agar (PDA) plates for ten days at 25 °C in the dark. (**C**) Colony morphology of strain GS-WW-224-VF cultured on PDA plates for ten days at 25 °C in the dark. (**D**) Average colony diameter of strains GS-WW-224 and GS-WW-224-VF incubated on PDA plates for seven days at 25 °C in the dark. (**E**) Average spore production in strains GS-WW-224 and GS-WW-224-VF cultured on PDA plates for seven days at 25 °C in the dark. (**F**) Average dry weight of mycelial biomass generated in strains GS-WW-224 and GS-WW-224-VF cultured in potato dextrose broth (PDB) for seven days at 25 °C in the dark. Asterisks indicate different levels of significant difference (*** *p* < 0.001) between the two strains as determined by paired t-test using GraphPad Prism version 8.0 software.

Purified virions of FaAV1 from *F. avenaceum* strain GS-WW-224 were observed to be isometric spherical and approximately 30 nm in diameter (Figure 3A). Their morphology was identical to the virions of *Alternaria alternata* virus-1 (AaV-1) [20]; however, virion size in FaAV1 was slightly smaller than the virions of AaV-1 [20], whose virions are approximately 33 nm in diameter. RT-PCR product was amplified from the RNA of the virions using FaAV1-specific primers and electrophoresed in a 1.0% (*w*/*v*) agarose gel, and the results of electrophoresis confirmed that FaAV1 was responsible for assembled virions extracted from *F. avenaceum* strain GS-WW-224.

### 3.3. Effect of FaAV1 on the Phenotype of Its Host Fungus F. avenaceum

The FaAV1-free strain GS-WW-224-VF was obtained using the hyphal tip detoxification method to eliminate FaAV1 from *F. avenaceum* strain GS-WW-224. The FaAV1-free status of GS-WW-224-VF was confirmed by 1.0% (*w*/*v*) gel electrophoretic profiles of dsRNA and RT-PCR analysis of dsRNA of FaAV1 using FaAV1-specific primers.

The colony surface of GS-WW-224 was felty (Figure 3B), while the colony surface of GS-WW-224-VF was flat (Figure 3C). No other significant differences in colony morphology were observed between strains GS-WW-224 and GS-WW-224-VF, both of which grew radially. The average colony growth rate of strain GS-WW-224 (10.58 mm/d) was similar to that of strain GS-WW-224-VF (10.66 mm/d) (Figure 3D). Additionally, no significant difference in average spore production was observed between strains GS-WW-224 (1.38 × 10^6^ spores/mL) and GS-WW-224-VF (1.66 × 10^6^ spores/mL) (Figure 3E). Average dry weight of the mycelial biomass generated by strain GS-WW-224 (1359.3 mg), however, was significantly higher than that of the mycelial biomass generated by strain GS-WW-224-VF (1093.1 mg) (Figure 3F).

**Figure 4:**
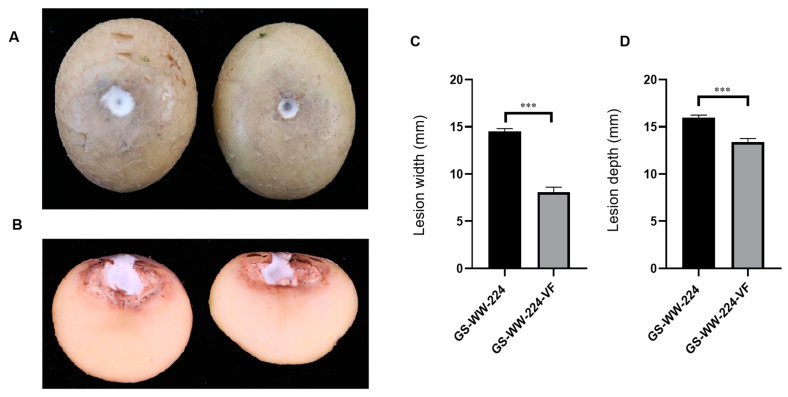
Virulence of *Fusarium avenaceum* strains GS-WW-224 and GS-WW-224-VF on potato tubers. (**A**) Representative photograph of dry rot symptoms caused by strains GS-WW-224 (on the left) and GS-WW-224-VF (on the right) on potato tubers at seven days post inoculation. (**B**) Representative photograph of a longitudinal section through a dry rot lesion caused by strains GS-WW-224 (on the left) and GS-WW-224-VF (on the right) on potato tubers. (**C**) Width of lesions on potato tubers infected by strains GS-WW-224 and GS-WW-224-VF. (**D**) Depth of lesions on potato tubers infected by strains GS-WW-224 and GS-WW-224-VF. Asterisks indicate different levels of significant difference (*** *p* < 0.001) between the two strains as determined by paired t-test using GraphPad Prism version 8.0 software.

Both GS-WW-224 and GS-WW-224-VF formed a white colony on the wounded surface of potato tubers (Figure 4A). Notably, the width and the depth (14.5 mm and 16.0 mm, respectively) of the lesions on potato tubers caused by strain GS-WW-224 were significantly greater than the width and the depth (8.10 mm and 13.4 mm, respectively) of lesions on potato tubers caused by strain GS-WW-224-VF (Figure 4B–D). These data indicate that FaAV1 infection confers hypervirulence on its host fungus *F. avenaceum* strain GS-WW-224.

### 3.4. Horizontal Transmission of FaAV1 and Transfection of Purified Virions

**Figure 5:**
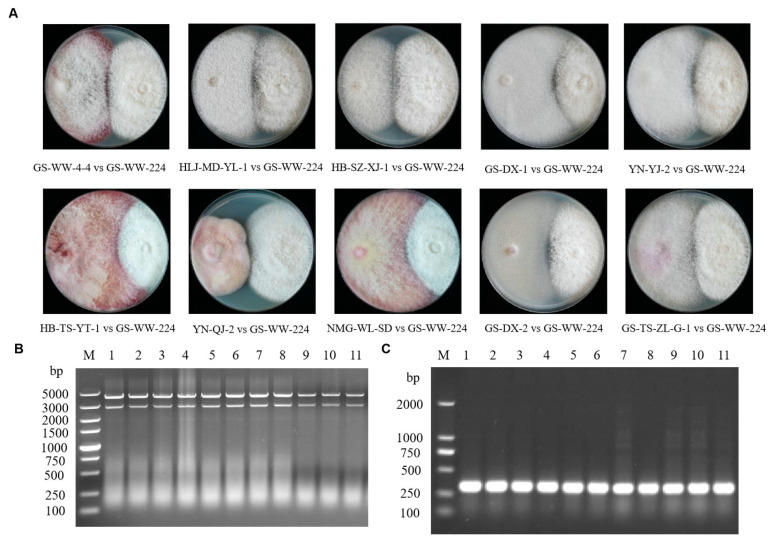
Horizontal transmission of the mycovirus *Fusarium avenaceum* alternavirus 1 (FaAV1) through hyphal contact in paired (donor-recipient) cultures. (**A**) Colonies formed in paired cultures of *Fusarium avenaceum* strain GS-WW-224 (donor strain, on the right) and each strain (recipient strain, on the left) obtained from ten other species of *Fusarium*. (**B**) Confirmation of the presence of FaAV1 in the derivative strains of the ten other species of *Fusarium* by 1.0% (*w*/*v*) agarose gel electrophoretic profiles of double-stranded RNA (dsRNA). Lane M: DNA molecular marker DL 5000; lane 1: The dsRNA extracted from *F. acuminatum* strain GS-WW-4-4; lane 2: The dsRNA extracted from *F. brachygibbosum* strain HLJ-MD-YL-1; lane 3: The dsRNA extracted from *F. equiseti* strain HB-SZ-XJ-1; lane 4: The dsRNA extracted from *F. flocciferum* strain GS-DX-1; lane 5: The dsRNA extracted from *F. graminearum* strain YN-YJ-2; lane 6: The dsRNA extracted from *F. oxysporum* strain HB-TS-YT-1; lane 7: The dsRNA extracted from *F. proliferatum* strain YN-QJ-2; lane 8: The dsRNA extracted from *F. redolens* strain NMG-WL-SD; lane 9: The dsRNA extracted from *F. sambucinum* strain GS-DX-2; lane 10: The dsRNA extracted from *F. solani* strain GS-TS-ZL-G-1; lane 11: The dsRNA extracted *F. avenaceum* strain GS-WW-224. (**C**) Confirmation of the presence of FaAV1 in the derivative strains of the ten other species of *Fusarium* by reverse transcription-polymerase chain reaction (RT-PCR) analysis of dsRNA using FaAV1-specific primers (dsRNA2-gap-1F and dsRNA2-gap-1R) in 1.0% (*w*/*v*) agarose gel electrophoresis. Lane M: DNA molecular marker DL 2000; lane 1: Products of RT-PCR from *F. acuminatum* strain GS-WW-4-4; lane 2: Products of RT-PCR from *F. brachygibbosum* strain HLJ-MD-YL-1; lane 3: Products of RT-PCR from *F. equiseti* strain HB-SZ-XJ-1; lane 4: Products of RT-PCR from *F. flocciferum* strain GS-DX-1; lane 5: Products of RT-PCR from *F. graminearum* strain YN-YJ-2; lane 6: Products of RT-PCR from *F. oxysporum* strain HB-TS-YT-1; lane 7: Products of RT-PCR from *F. proliferatum* strain YN-QJ-2; lane 8: Products of RT-PCR from *F. redolens* strain NMG-WL-SD; lane 9: Products of RT-PCR from *F. sambucinum* strain GS-DX-2; lane 10: Products of RT-PCR from *F. solani* strain GS-TS-ZL-G-1; lane 11: Products of RT-PCR from *F. avenaceum* strain GS-WW-224.

Paired cultures of donor strain GS-WW-224 and each of the recipient strains of the ten other species of *Fusarium* carrying a hygromycin B phosphotransferase gene were plated on PDA plates not amended with 50 µg/mL of hygromycin to conduct the horizontal transmission assays (Figure 5A). Derivative strains obtained from the hyphal interaction zone were then cultured on fresh PDA plates containing hygromycin, on which only the recipient strains of the ten other species of *Fusarium* carrying the hygromycin B phosphotransferase gene could grow. Analysis of the derivative strains indicated that all the ten recipient strains of *Fusarium* carried the mycovirus FaAV1, as confirmed by 1.0% (*w*/*v*) gel electrophoretic profiles of dsRNA (Figure 5B) and the RT-PCR detection of dsRNA of FaAV1 (Figure 5C) using FaAV1-specific primers. These data indicate that FaAV1 can be transmitted horizontally from *F. avenaceum* to at least ten other species of *Fusarium* by hyphal contact in paired donor-recipient cultures.

FaAV1 dsRNA was identified by 1.0% (*w*/*v*) gel electrophoretic profiles of dsRNA in all of the subcultures derived from virion-transfected, regenerated mycelia of the ten other species of *Fusarium*. Viral transfection was further verified by RT-PCR detection of dsRNA of FaAV1 in the ten derived strains from the ten other species of *Fusarium* using FaAV1-specific primers. FaAV1 was readily detected in all ten of the transfected strains of the ten other species of *Fusarium* after being subcultured three times, indicating that the dsRNA of FaAV1 was stable in each subculture. Collectively, these results indicate that purified virions of FaAV1 can transfect the hyphae of at least ten other species of *Fusarium*.

## 4. Discussion

In the present study, we identified and characterized a novel mycovirus with a dsRNA genome in *F. avenaceum* strain GS-WW-224 causing potato dry rot. The mycovirus was designated as *Fusarium avenaceum* alternavirus 1 (FaAV1) in the proposed family Alternaviridae. The FaAV1-free strain GS-WW-224-VF was successfully derived from the FaAV1-infected strain GS-WW-224 using hyphal tipping methodology. No significant differences were observed in colony morphology and spore production between the strains GS-WW-224 and GS-WW-224-VF. The dry weight of the mycelial biomass and the virulence on potato tubers of GS-WW-224, however, were significantly greater than the same parameters in strain GS-WW-224-VF. We also demonstrated that FaAV1 could overcome vegetative incompatibility barriers and be transmitted horizontally from GS-WW-224 to ten other species of *Fusarium*. Purified virions of FaAV1 could also infect the hyphal fragments of at least ten other species of *Fusarium*. To the best of our knowledge, this is the first report of an alternavirus infecting *F. avenaceum*.

At least forty-seven mycoviruses and their full-length genomes have been reported in eighteen species of *Fusarium* [27,52]; however, no mycoviruses have been identified in *F. avenaceum*, one of the main phytopathogenic species of *Fusarium* causing potato dry rot. In the present study, FaAV1 was extracted from *F. avenaceum* strain GS-WW-224 and identified to be a new alternavirus, which is the first time that an alternavirus has been identified in *F. avenaceum*. Thus far, eleven mycoviruses have been designated as members of the proposed family Alternaviridae [7,19,20,21,22,23,24,25,26,27,28,29]. Among them, FiAV1 [7], FpAV1 [23], FgAV1 [24], AheAV1 [25], and CcAV1 [28] contain three dsRNA segments, while AsV341 [19], AaV1 [20,21], AfV [22], FoAV1 [26], FsAV1 [27], and SlAV1 [29] contain four dsRNA segments. The genomic composition of FaAV1 identified in the present study, however, only contains two dsRNA segments. In this regard, the number of dsRNA segments in members of the family *Chrysoviridae* has been reported to range from four to five [53,54,55,56]. Therefore, we hypothesize that the number of dsRNA segments in different members within the proposed family Alternaviridae may also vary and range from two to four.

*Fusarium* graminearum virus-DK21 (FgV1-DK21) was the first mycovirus, among the mycoviruses infecting *Fusarium*, reported to cause pronounced morphological changes in its host, *F. graminearum*. The altered phenotypes included reduced mycelial growth, increased pigmentation, reduced virulence on wheat, and decreased production of trichothecene mycotoxins [57]. *F. graminearum* strains infected with *Fusarium* graminearum virus-ch9 (Fgv-ch9) [58] exhibit a reduced rate of mycelial growth and conidial production, abnormal colony morphology, and disorganized cytoplasm, as well as reduced virulence. *Fusarium* equiseti partitivirus 1 (FePV1) has been reported to confer hypovirulence to its host, *Fusarium equiseti*, including a lower growth rate, biomass production, and virulence on tomato [16]. *Fusarium* oxysporum f. sp. dianthi mycovirus 1 (FodHV1), *Fusarium* oxysporum ourmia-like virus 1 (FoOuLV1), and *Fusarium* oxysporum alternavirus 1 (FoAV1), which were all isolated from *F. oxysporum*, also confer hypovirulent characteristics on their host fungi [8,26,59]. In the current study, no significant differences were observed in colony morphology and spore production between strains GS-WW-224 and GS-WW-224-VF. Relative to strain GS-WW-224-VF, however, the dry weight of the mycelial biomass was higher in strain GS-WW-224 than it was in GS-WW-224-VF. The virulence of strain GS-WW-224 infected with FaAV1 was also greater, relative to strain GS-WW-224-VF, suggesting that FaAV1 confers hypervirulence to its host *F. avenaceum*. Notably, this is the first report of a mycovirus potentially conveying increased virulence in a species within the genus *Fusarium*.

Transmission of mycoviruses through hyphal anastomosis naturally occurs between individuals of closely related vegetative compatibility groups [45]. Therefore, the host range of mycoviruses is commonly thought to be limited to a single species. *Sclerotinia sclerotiorum* partitivirus 1 (SsPV1/WF-1), however, was readily transmitted horizontally through hyphal contact between different vegetative compatibility groups in *S. sclerotiorum* [45]. *Alternaria alternata* botybirnavirus 1 (AaBRV1) was transmitted horizontally from the AaBRV1-AT1-infected *Alternaria tenuissima* strain TJ-NH-51S-4 to the AaBRV1-AT1-free strain XJ-BZ-5-1 (another strain of *A. tenuissima*) through hyphal contact in paired cultures [60]. Interspecies transmission of mycoviruses between closely-related species has also been documented. The first report of interspecific transmission of dsRNA was the transmission of *Sclerotinia sclerotiorum* virus 1 (Ssv1) from *Sclerotinia sclerotiorum* to *S. minor* through hyphal anastomosis [61]. *Fusarium* poae virus 1-Fa (FpV1-Fa) was also reported to be transferred from *F. asiaticum* to *F. poae* and *F. tricinctum* through hyphal anastomosis [62]. FgV1-DK21 virus was transmitted horizontally from *F. boothii* to *F. graminearum*, *F. asiaticum*, *F. oxysporum* f. sp. *lycopersici*, and *Cryphonectria parasitica* via protoplast fusion [63]. In our study, FaAV1 was successfully transmitted horizontally from *F. avenaceum* to ten other species of *Fusarium* through hyphal contact in paired donor-recipient cultures. This infers that FaAV1 has the ability to overcome the vegetative incompatibility barriers that exist between different species of *Fusarium*.

Virion-based transfection techniques are commonly used in experimental studies to extend the host range of mycoviruses. Rosellinia necatrix partitivirus 1-W8 (RnPV1-W8) and Rosellinia necatrix mycoreovirus 3 (MyRV3) were transmitted from *Rosellinia necatrix* to *Diaporthe* sp., *Cryphonectria parasitica*, *Valsa ceratosperma*, and *Glomerella cingulate* by inoculation of protoplasts with virions, thus, extending the experimental host range of RnPV1-W8 and MyRV3 [64]. Virions of *Sclerotinia sclerotiorum* hypovirulence-associated DNA virus 1 (SsHADV-1) isolated from an infected host (*Sclerotinia sclerotiorum*) can directly infect the hyphae of virus-free strains of *S. sclerotiorum*, *S. minor*, and *S. nivalis*, when virions are applied to intact hyphae grown on PDA plates or to hyphal fragments [65]. In the present study, virions of FaAV1 were used to directly infect wounded hyphae (referred to as hyphal fragments) instead of protoplasts, and results confirmed that virions of FaAV1 can directly infect hyphal fragments of the ten other species of *Fusarium*. Notably, however, we did not determine if virions of FaAV1 could directly infect intact hyphae of the ten other species of *Fusarium* grown on PDA plates.

The present study was the first to identify a new alternavirus, namely FaAV1, infecting *F. avenaceum* in the proposed family Alternaviridae and initially characterize the effect of FaAV1 infection on the phenotype of its host fungus *F. avenaceum*. Our study also confirmed the ability of FaAV1 to overcome incompatibility barriers and undergo horizontal transmission from *F. avenaceum* to ten other species of *Fusarium*. Furthermore, the direct transfection of virions of FaAV1 through wounded hyphae in ten other species of *Fusarium* was testified in this study. An experimental system was established in our study that can be utilized to study the interaction between alternaviruses and their host fungi. In summary, our results provided a foundation for the further study of alternaviruses in the proposed family Alternaviridae. Comparative transcriptome analysis of FaAV1-infected and FaAV1-free *F. avenaceum* strains will be conducted in future studies to identify differentially expressed genes (DEGs) between the two strains, and the potential functional impact of DEGs resulting from FaAV1 infection on the biological properties of *F. avenaceum*.

## Figures and Tables

**Table 1:** *Fusarium* strains used in this study.

Strain Code	Geographical Origin	Species of *Fusarium*	*Fusarium avenaceum* AlternaVirus 1 (FaAV1)	Hygromycin B PhosphotransferAse Gene
GS-WW-224	Wuwei city, Gansu province, China	*Fusarium avenaceum*	Positive	Not Labeled
GS-WW-4-4	Wuwei city, Gansu province, China	*F. acuminatum*	Negative	Labeled
HLJ-MD-YL-1	Mudanjiang city, Heilongjiang province, China	*F. brachygibbosum*	Negative	Labeled
HB-SZ-XJ-1	Jingzhou city, Hubei province, China	*F. equiseti*	Negative	Labeled
GS-DX-1	Dingxi city, Gansu province, China	*F. flocciferum*	Negative	Labeled
YN-YJ-2	Dehong Dai and Jingpo autonomous prefecture, Yunan province, China	*F. graminearum*	Negative	Labeled
HB-TS-YT-1	Tangshan city, Hebei province, China	*F. oxysporum*	Negative	Labeled
YN-QJ-2	Qujing city, Yunnan province, China	*F. proliferatum*	Negative	Labeled
NMG-WL-SD	Ulanqab city, Inner Mongolia autonomous region, China	*F. redolens*	Negative	Labeled
GS-DX-2	Dingxi city, Gansu province, China	*F. sambucinum*	Negative	Labeled
GS-TS-ZL-G-1	Tianshui city, Gansu province, China	*F. solani*	Negative	Labeled

**Table 2:** Amino acid sequence identity of RNA dependent RNA polymerase (RdRp) and hypothetical protein (HP) of *Fusarium avenaceum* alternavirus 1 (FaAV1) to other alternaviruses.

FaAV1	Names of Other Alternaviruses	Accession Number	Query Cover	E-Value	Identity (%)	Length (Amino Acid)
RdRp domain	*Fusarium* poae alternavirus 1	YP_009272952.1	100%	0	84.42	1123
	*Fusarium* graminearum alternavirus 1	YP_009449439.1	100%	0	84.33	1123
	*Fusarium* incarnatum alternavirus 1	AYJ09265.1	100%	0	83.26	1123
	Aspergillus foetidus dsRNA mycovirus	YP_007353985.1	100%	0	45.94	1124
	Aspergillus mycovirus 341	ABX79997.1	95%	0	47.41	1124
	Aspergillus heteromorphus alternavirus 1	AZT88575.1	100%	0	45.70	1124
	Alternaria alternata virus 1	YP_001976142.1	100%	0	35.69	1149
	*Fusarium* oxysporum alternavirus 1	QYY49562.2	60%	0	47.60	744
	Stemphylium lycopersici alternavirus 1	YP_009551660.1	98%	0	34.28	1148
	*Fusarium* solani alternavirus 1	UQZ09636.1	96%	0	46.02	1128
	Cordyceps chanhua alternavirus 1	UPH33984.1	95%	0	46.15	1127
HP domain	*Fusarium* graminearum alternavirus 1	AUI80777.1	100%	0	80.89	743
	*Fusarium* poae alternavirus 1	YP_009272950.1	99%	0	80.86	743
	*Fusarium* incarnatum alternavirus 1	AYJ09267.1	100%	0	77.25	739
	Aspergillus foetidus dsRNA mycovirus	YP_007353983.1	94%	5 × 10^−51^	28.38	726
	Aspergillus heteromorphus alternavirus 1	AZT88577.1	94%	5 × 10^−43^	28.24	727
	*Fusarium* oxysporum alternavirus 1	QYY49564.1	47%	4 × 10^−41^	35.89	487
	Alternaria alternata virus 1	YP_001976151.1	35%	1 × 10^−9^	30.15	759
	*Fusarium* solani alternavirus 1	UQZ09637.1	72%	2 × 10^−40^	38.63	831
	Cordyceps chanhua alternavirus 1	UPH33985.1	92%	3 × 10^−55^	30.11	831
	Stemphylium lycopersici alternavirus 1	YP_009551663.1	32%	1 × 10^−6^	30.77	756

## Data Availability

The whole genome sequence of *Fusarium avenaceum* alternavirus 1 (FaAV1) has been deposited in the GenBank database under the accession numbers OK556479 and OK556480.

## Supplementary Materials

The following are available online at https://www.mdpi.com/article/10.3390/v15010145/s1, Table S1: Primers used in this study to determine the complete genome sequence of the mycovirus, *Fusarium avenaceum* alternavirus 1 (FaAV1).

## References

[1] Ghabrial S.A., Suzuki N. (2009). Viruses of plant pathogenic fungi. Annu. Rev. Phytopathol..

[2] Kotta-Loizou I. (2021). Mycoviruses and their role in fungal pathogenesis. Curr. Opin. Microbiol..

[3] Abdoulaye A.H., Foda M.F., Kotta-Loizou I. (2019). Viruses infecting the plant pathogenic fungus *Rhizoctonia solani*. Viruses.

[4] Jia H.X., Dong K.L., Zhou L.L., Wang G.P., Hong N., Jiang D.H., Xu W.X. (2017). A dsRNA virus with filamentous viral particles. Nat. Commun..

[5] Kanhayuwa L., Kotta-Loizou I., Oezkan S., Gunning A.P., Coutts R.H.A. (2015). A novel mycovirus from *Aspergillus fumigatus* contains four unique dsRNAs as its genome and is infectious as dsRNA. Proc. Natl. Acad. Sci. USA..

[6] Kotta-Loizou I., Coutts R.H.A. (2017). Studies on the virome of the entomopathogenic fungus *Beauveria bassiana* reveal novel dsRNA elements and mild hypervirulence. PLoS Pathog..

[7] Zhang X.T., Xie Y., Zhang F., Sun H.J., Zhai Y.T., Zhang S.B., Yuan H.X., Zhou L., Gao F., Li H.L. (2019). Complete genome sequence of an alternavirus from the phytopathogenic fungus *Fusarium incarnatum*. Arch. Virol..

[8] Zhao Y., Zhang Y.Y., Wan X.R., She Y.Y., Li M., Xi H.J., Xie J.T., Wen C.Y. (2020). A novel ourmia-like mycovirus confers hypovirulence-associated traits on *Fusarium oxysporum*. Front. Microbiol..

[9] Darissa O., Willingmann P., Schäfer W., Adam G. (2011). A novel double-stranded RNA mycovirus from *Fusarium graminearum*: Nucleic acid sequence and genomic structure. Arch. Virol..

[10] Chen X.G., He H., Yang X.F., Zeng H.M., Qiu D.W., Guo L.H. (2016). The complete genome sequence of a novel *Fusarium* graminearum RNA virus in a new proposed family within the order *Tymovirales*. Arch. Virol..

[11] Wang L., Zhang J.Z., Zhang H.L., Qiu D.W., Guo L.H. (2016). Two novel relative double-stranded RNA mycoviruses infecting *Fusarium poae* strain SX63. Int. J. Mol. Sci..

[12] Zhang L.H., Chen X.G., Bhattacharjee P., Shi Y., Guo L.H., Wang S.C. (2020). Molecular characterization of a novel strain of *Fusarium* graminearum virus 1 infecting *Fusarium graminearum*. Viruses.

[13] Li P.F., Wang S.C., Zhang L.H., Qiu D.W., Zhou X.P., Guo L.H. (2020). A tripartite ssDNA mycovirus from a plant pathogenic fungus is infectious as cloned DNA and purified virions. Sci. Adv..

[14] Xie Y., Wang Z.F., Li K., Liu D.W., Jia Y.F., Gao F., Dai J.L., Zhang S.B., Zhang X.T., Li H.L. (2022). A megabirnavirus alleviates the pathogenicity of *Fusarium pseudograminearum* to wheat. Phytopathology.

[15] Mizutani Y., Abraham A., Uesaka K., Kondo H., Suga H., Suzuki N., Chiba S. (2018). Novel mitoviruses and a unique tymo-like virus in hypovirulent and virulent strains of the *Fusarium* head blight fungus, *Fusarium boothii*. Viruses.

[16] Mahillon M., Decroës A., Caulier S., Tiendrebeogo A., Legrève A., Bragard C. (2021). Genomic and biological characterization of a novel partitivirus infecting *Fusarium equiseti*. Virus Res..

[17] Mahillon M., Decroës A., Liénard C., Bragard C., Legrève A. (2019). Full genome sequence of a new polymycovirus infecting *Fusarium redolens*. Arch. Virol..

[18] Li W., Xia Y.L., Zhang H.T., Zhang X., Chen H.G. (2019). A victorivirus from *Fusarium asiaticum*, the pathogen of *Fusarium* head blight in China. Arch. Virol..

[19] Hammond T.M., Andrewski M.D., Roossinck M.J., Keller N.P. (2008). *Aspergillus* mycoviruses are targets and suppressors of RNA silencing. Eukaryot. Cell.

[20] Aoki N., Moriyama H., Kodama M., Arie T., Teraoka T., Fukuhara T. (2009). A novel mycovirus associated with four double-stranded RNAs affects host fungal growth in *Alternaria alternata*. Virus Res..

[21] Wu C.F., Aoki N., Takeshita N., Fukuhara T., Chiura H.X., Arie T., Kotta-Loizou I., Okada R., Komatsu K., Moriyama H. (2021). Unique terminal regions and specific deletions of the segmented double-stranded RNA genome of *Alternaria alternata* virus 1, in the proposed family Alternaviridae. Front. Microbiol..

[22] Kozlakidis Z., Herrero N., Ozkan S., Kanhayuwa L., Jamal A., Bhatti M.F., Coutts R.H. (2013). Sequence determination of a quadripartite dsRNA virus isolated from *Aspergillus foetidus*. Arch. Virol..

[23] Osaki H., Sasaki A., Nomiyama K., Tomioka K. (2016). Multiple virus infection in a single strain of *Fusarium poae* shown by deep sequencing. Virus Genes.

[24] He H., Chen X.G., Li P.F., Qiu D.W., Guo L.H. (2018). Complete genome sequence of a *Fusarium* graminearum double-stranded RNA virus in newly proposed family, Alternaviridae. Genome Announc..

[25] Gilbert K.B., Holcomb E.E., Allscheid R.L., Carrington J.C. (2019). Hiding in plain sight: New virus genomes discovered via a systematic analysis of fungal public transcriptomes. PLoS ONE.

[26] Wen C.Y., Wan X.R., Zhang Y.Y., Du H.Y., Wei C.X., Zhong R.R., Zhang H., Shi Y., Xie J.T., Fu Y.P. (2021). Molecular characterization of the first alternavirus identified in *Fusarium oxysporum*. Viruses.

[27] Lutz T., Japić E., Bien S., Langer G.J., Heinze C. (2022). Characterization of a novel alternavirus infecting the fungal pathogen *Fusarium solani*. Virus Res..

[28] Zhang Y.X., Shi N.J., Wang P., Zhu Q.Y., Yang G.G., Huang B. (2022). Molecular characterization of a novel alternavirus infecting the entomopathogenic fungus *Cordyceps chanhua*. Arch. Virol..

[29] Liu H., Wang H., Liao X.L., Gao B., Lu X., Sun D., Gong W., Zhong J., Zhu H., Pan X. (2022). Mycoviral gene integration converts a plant pathogenic fungus into a biocontrol agent. Proc. Natl. Acad. Sci. USA..

[30] Tiwari R.K., Kumar R., Sharma S., Sagar V., Aggarwal R., Naga K.C., Lal M.K., Chourasia K.N., Kumar D., Kumar M. (2020). Potato dry rot disease: Current status, pathogenomics and management. 3 Biotech.

[31] Rampersad S.N. (2020). Pathogenomics and management of *Fusarium* diseases in plants. Pathogens.

[32] Venter S.L., Steyn P.J. (1998). Correlation between fusaric acid production and virulence of isolates of *Fusarium oxysporum* that causes potato dry rot in South Africa. Potato Res..

[33] Stevenson W.R., Loria R., Franc G.D., Weingartner D.P. (2001). Compendium of Potato Diseases.

[34] Choiseul J., Allen L., Carnegie S.F. (2006). Fungi causing dry tuber rots of seed potatoes in storage in Scotland. Potato Res..

[35] Gachango E., Hanson L.E., Rojas A., Hao J.J., Kirk W.W. (2012). *Fusarium* spp. causing dry rot of seed potato tubers in Michigan and their sensitivity to fungicides. Plant Dis..

[36] Osaki H., Sasaki A., Nomiyama K., Sekiguchi H., Tomioka K., Takehara T. (2015). Isolation and characterization of two mitoviruses and a putative alphapartitivirus from *Fusarium* spp.. Virus Genes.

[37] Zhang X.F., Li S.W., Ma Z.H., Cai Q.N., Zhou T., Wu X.H. (2022). Complete genome sequence of a novel mitovirus isolated from the fungus *Fusarium equiseti* causing potato dry rot. Arch. Virol..

[38] Yaegashi H., Kanematsu S., Ito T. (2012). Molecular characterization of a new hypovirus infecting a phytopathogenic fungus, *Valsa ceratosperma*. Virus Res..

[39] Li Y.T., Li S.W., Liang Z.J., Cai Q.N., Zhou T., Zhao C., Wu X.H. (2022). RNA-seq analysis of *Rhizoctonia solani* AG-4HGI strain BJ-1H infected by a new viral strain of Rhizoctonia solani partitivirus 2 reveals a potential mechanism for hypovirulence. Phytopathology.

[40] Morris T.J., Dodds J.A. (1979). Isolation and analysis of double-stranded RNA from virus-infected plant and fungal tissue. Phytopathology.

[41] Ma G.P., Liang Z.J., Hua H.H., Zhou T., Wu X.H. (2019). Complete genome sequence of a new botybirnavirus isolated from a phytopathogenic *Alternaria alternata* in China. Arch. Virol..

[42] Thompson J.D., Gibson T.J., Plewniak F., Jeanmougin F., Higgins D.G. (1997). The CLUSTAL_X windows interface: Flexible strategies for multiple sequence alignment aided by quality analysis tools. Nucleic Acids Res..

[43] Tamura K., Stecher G., Peterson D., Filipski A., Kumar S. (2013). MEGA6: Molecular evolutionary genetics analysis version 6.0. Mol. Biol. Evol..

[44] Chiba S., Salaipeth L., Lin Y.H., Sasaki A., Kanematsu S., Suzuki N. (2009). A novel bipartite double-stranded RNA mycovirus from the white root rot fungus *Rosellinia necatrix*: Molecular and biological characterization, taxonomic considerations, and potential for biological control. J. Virol..

[45] Xiao X.Q., Cheng J.S., Tang J.H., Fu Y.P., Jiang D.H., Baker T.S., Ghabrial S.A., Xie J.T. (2014). A novel partitivirus that confers hypovirulence on plant pathogenic fungi. J. Virol..

[46] Liu J., Zhang X.F., Kennedy J.F., Jiang M.G., Cai Q.N., Wu X.H. (2019). Chitosan induces resistance to tuber rot in stored potato caused by *Alternaria tenuissima*. Int. J. Biol. Macromol..

[47] Aprasad K.S., Bateman G.L., Read P.J. (1997). Variation in pathogenicity on potato tubers and sensitivity to thiabendazole of the dry rot fungus *Fusarium avenaceum*. Potato Res..

[48] Hou Z.M., Xue C., Peng Y.L., Katan T., Kistler H.C., Xu J.R. (2002). A mitogen-activated protein kinase gene (*MGV1*) in *Fusarium graminearum* is required for female fertility, heterokaryon formation, and plant infection. Mol. Plant Microbe In..

[49] Kamaruzzaman M., He G., Wu M., Zhang J., Yang L., Chen W., Li G. (2019). A novel partitivirus in the hypovirulent isolate QT5-19 of the plant pathogenic fungus *Botrytis cinerea*. Viruses.

[50] Li H., Bian R., Liu Q., Yang L., Pang T., Salaipeth L., Andika I.B., Kondo H., Sun L. (2019). Identification of a novel hypovirulence-inducing hypovirus from *Alternaria alternata*. Front. Microbiol..

[51] Sasaki A., Kanematsu S., Onoue M., Oyama Y., Yoshida K. (2006). Infection of *Rosellinia necatrix* with purified viral particles of a member of *Partitiviridae* (RnPV1-W8). Arch. Virol..

[52] Li P.F., Bhattacharjee P., Wang S.C., Zhang L.H., Ahmed I., Guo L.H. (2019). Mycoviruses in *Fusarium* species: An update. Front. Cell Infect. Mi..

[53] Urayama S., Ohta T., Onozuka N., Sakoda H., Fukuhara T., Arie T., Teraoka T., Moriyama H. (2012). Characterization of Magnaporthe oryzae chrysovirus 1 structural proteins and their expression in *Saccharomyces cerevisiae*. J. Virol..

[54] Komatsu K., Urayama S., Katoh Y., Fuji S., Hase S., Fukuhara T., Arie T., Teraoka T., Moriyama H. (2016). Detection of Magnaporthe oryzae chrysovirus 1 in Japan and establishment of a rapid, sensitive and direct diagnostic method based on reverse transcription loop-mediated isothermal amplification. Arch. Virol..

[55] Jamal A., Bignell E.M., Coutts R.H. (2010). Complete nucleotide sequences of four dsRNAs associated with a new chrysovirus infecting *Aspergillus fumigatus*. Virus Res..

[56] Wang L.P., Jiang J.J., Wang Y.F., Hong N., Zhang F.P., Xu W.X., Wang G.P. (2014). Hypovirulence of the phytopathogenic fungus *Botryosphaeria dothidea*: Association with a coinfecting chrysovirus and a partitivirus. J. Virol..

[57] Chu Y.M., Jeon J.J., Yea S.J., Kim Y.H., Yun S.H., Lee Y.W., Kim K.H. (2002). Double-stranded RNA mycovirus from *Fusarium graminearum*. Appl. Environ. Microb..

[58] Darissa O., Adam G., Schäfer W. (2012). A dsRNA mycovirus causes hypovirulence of *Fusarium graminearum* to wheat and maize. Eur. J. Plant Pathol..

[59] Torres-Trenas A., Cañizares M.C., García-Pedrajas M.D., Pérez-Artés E. (2020). Molecular and biological characterization of the first hypovirus identified in *Fusarium oxysporum*. Front. Microbiol..

[60] Liang Z.J., Hua H.H., Wu C.Y., Zhou T., Wu X.H. (2022). A botybirnavirus isolated from *Alternaria tenuissima* confers hypervirulence and decreased sensitivity of its host fungus to difenoconazole. Viruses.

[61] Melzer M.S., Ikeda S.S., Boland G.J. (2002). Interspecific transmission of double-stranded RNA and hypovirulence from *Sclerotinia sclerotiorum* to *S. minor*. Phytopathology.

[62] Song X.S., Sun Y.D., Gao J., Gu K.X., Hou Y.P., Wang J.X., Zhou M.G. (2022). Extending the host range of *Fusarium* poae virus 1 from *Fusarium poae* to other *Fusarium* species in the field. Viruses.

[63] Lee K.M., Yu J., Son M., Lee Y.W., Kim K.H. (2011). Transmission of *Fusarium* boothii mycovirus via protoplast fusion causes hypovirulence in other phytopathogenic fungi. PLoS ONE.

[64] Kanematsu S., Sasaki A., Onoue M., Oikawa Y., Ito T. (2010). Extending the fungal host range of a partitivirus and a mycoreovirus from *Rosellinia necatrix* by inoculation of protoplasts with virus particles. Phytopathology.

[65] Yu X., Li B., Fu Y.P., Xie J.T., Cheng J.S., Ghabrial S.A., Li G.Q., Yi X.H., Jiang D.H. (2013). Extracellular transmission of a DNA mycovirus and its use as a natural fungicide. Proc. Natl. Acad. Sci. USA..

