# Supplementary material for: Characterization of the First Alternavirus Identified in Fusarium avenaceum, the Causal Agent of Potato Dry Rot
Source: Viruses. 2023 Jan 2;15(1):145. doi: 10.3390/v15010145 (PMC9864086; doi:10.3390/v15010145)
Supplement: Supplementary file 1 [file viruses-15-00145-s001.zip › Supplementary Table S1.pdf]

Supplementary Table S1. Primers used in this study to determine the complete genome sequence of the mycovirus, Fusarium avenaceum alternavirus 1 (FaAV1).

Primer name	Sequence (5'-3')
PC3-T7 loop	p-GGATCCCGGGAATTCGGTAATACGACTCACTATATTTTT ATAGTGAGTCGTATTA-OH
PC2	CCGAATTCCCGGGATCC
RACE3 RT	CGATCGATCATGATGCAATGCNNNNNN
RACE3	CGATCGATCATGATGCAATGC
dsRNA1-3end-1	GAAGGCGAGGTATCTATCAT
dsRNA1-3end-2	ATGGATAGGGTTGTGGGTCA
dsRNA1-3end-3	GGATAGGGTTGTGGGTCA
dsRNA1-5end-1	CCGACCTTAGGGTACTTG
dsRNA1-5end-2	GTAGCTTGAATACATCGAGGAG
dsRNA1-5end-3	CCAAGCTCCACGGCTCCACT
dsRNA1-gap-1F	CGAAAGGCATAGGATTGAC
dsRNA1-gap-1R	GAAGATGGTGAAGAGCCAGAGGAAG
dsRNA1-gap-2F	TGTTCTCGACAGCACCTCT
dsRNA1-gap-2R	ATCAGCGTATTTCCTCAATG
dsRNA1-gap-3F	CGACCTTAGGGTACTTGTTG
dsRNA1-gap-3R	CTGCTAGGGTAGTGTTGTGC
dsRNA1-gap-4F	AATCCGTGACCCACAACC
dsRNA1-gap-4R	AGAAGGAGATGATGGCAGAG
dsRNA2-3end-1	GACCTGCCTGTGCGTTGGA
dsRNA2-3end-2	TGCCTGTGCGTTGGAAGC
dsRNA2-3end-3	GACACGAGTGGGACGGACTT
dsRNA2-5end-1	TGAAGGTCAAGTCCGTCCCA
dsRNA2-5end-2	TCAGAAGGTTGAGGCGAAGC
dsRNA2-5end-3	TGTCTGCTGCGGGAGGTT
dsRNA2-gap-1F	GTCGTGAACGCTCGCACT

dsRNA2-gap-1R	GGAACGCAGTTGTGGAGG
dsRNA2-gap-2F	GTCGTGAACGCTCGCACT
dsRNA2-gap-2R	GGAACGCAGTTGTGGAGG
dsRNA2-gap-3F	GGTAACACCGAACTTGACTT
dsRNA2-gap-3R	TTGAACAACTTGAGCCTGTC
dsRNA2-gap-4F	GCATCAGCAGCTTCTCCAT
dsRNA2-gap-4R	CCAGCAGGAAAGCGACTC
